# Experience of Health Care Professionals Using Digital Tools in the Hospital: Qualitative Systematic Review

**DOI:** 10.2196/50357

**Published:** 2023-10-17

**Authors:** Marie Wosny, Livia Maria Strasser, Janna Hastings

**Affiliations:** 1 School of Medicine University of St Gallen (HSG) St Gallen Switzerland; 2 Institute for Implementation Science in Health Care University of Zurich (UZH) Zurich Switzerland

**Keywords:** health information technology, electronic health record, electronic medical records, clinical decision support, health care professionals, burnout, qualitative research

## Abstract

**Background:**

The digitalization of health care has many potential benefits, but it may also negatively impact health care professionals’ well-being. Burnout can, in part, result from inefficient work processes related to the suboptimal implementation and use of health information technologies. Although strategies to reduce stress and mitigate clinician burnout typically involve individual-based interventions, emerging evidence suggests that improving the experience of using health information technologies can have a notable impact.

**Objective:**

The aim of this systematic review was to collect evidence of the benefits and challenges associated with the use of digital tools in hospital settings with a particular focus on the experiences of health care professionals using these tools.

**Methods:**

We conducted a systematic literature review following the PRISMA (Preferred Reporting Items for Systematic Reviews and Meta-Analyses) guidelines to explore the experience of health care professionals with digital tools in hospital settings. Using a rigorous selection process to ensure the methodological quality and validity of the study results, we included qualitative studies with distinct data that described the experiences of physicians and nurses. A panel of 3 independent researchers performed iterative data analysis and identified thematic constructs.

**Results:**

Of the 1175 unique primary studies, we identified 17 (1.45%) publications that focused on health care professionals’ experiences with various digital tools in their day-to-day practice. Of the 17 studies, 10 (59%) focused on clinical decision support tools, followed by 6 (35%) studies focusing on electronic health records and 1 (6%) on a remote patient-monitoring tool. We propose a theoretical framework for understanding the complex interplay between the use of digital tools, experience, and outcomes. We identified 6 constructs that encompass the positive and negative experiences of health care professionals when using digital tools, along with moderators and outcomes. Positive experiences included feeling confident, responsible, and satisfied, whereas negative experiences included frustration, feeling overwhelmed, and feeling frightened. Positive moderators that may reinforce the use of digital tools included sufficient training and adequate workflow integration, whereas negative moderators comprised unfavorable social structures and the lack of training. Positive outcomes included improved patient care and increased workflow efficiency, whereas negative outcomes included increased workload, increased safety risks, and issues with information quality.

**Conclusions:**

Although positive and negative outcomes and moderators that may affect the use of digital tools were commonly reported, the experiences of health care professionals, such as their thoughts and emotions, were less frequently discussed. On the basis of this finding, this study highlights the need for further research specifically targeting experiences as an important mediator of clinician well-being. It also emphasizes the importance of considering differences in the nature of specific tools as well as the profession and role of individual users.

**Trial Registration:**

PROSPERO CRD42023393883; https://tinyurl.com/2htpzzxj

## Introduction

### Background

The digitalization of the health care industry and hospitals aims to enhance the quality of patient care [1], increase operational efficiency [2], and reduce health care expenditure [3]. The use of digital technologies in health care settings has gained momentum in recent years with the introduction of various digital tools, including electronic health records (EHRs) [4], clinical decision support (CDS) tools [5], artificial intelligence (AI) applications [6], telemedicine [7], wearable devices [8], and health apps [9], which hold great potential to transform and revolutionize the delivery of health care services [10]. This trend is expected to accelerate with recent advances in AI technologies for language [11-14].

Despite the potential benefits, digitalization in health care raises concern about the well-being of health care professionals (HCPs) [15,16]*.* Previous research has demonstrated that suboptimal use of health information technologies and inefficient work processes can be associated with burnout, leading to feelings of frustration and reduced job satisfaction among HCPs [17,18]. In 2022, a study with >13,000 participants revealed that 48% of physicians working in hospitals reported feeling burned out, with the use of EHR cited as a main factor by 28% of respondents [19]. Similar findings, including the association of EHR design and use factors with clinicians’ stress and burnout, have been reported [20,21]. Burnout is a prolonged response to chronic work-related stress and is characterized by exhaustion, cynicism, and inefficacy and is influenced by both individual and organizational factors [22]. Clinician burnout can negatively affect the quality of care and can result in a range of negative consequences, including dysfunctional relationships with colleagues, self-medication or substance abuse, depression, and even suicide [23].

This issue becomes even more significant when considering physician burnout, as it is associated with physicians leaving clinical practice, consequently impacting a country’s health care system [24]. The loss of physicians from the workforce is an escalating problem in numerous countries, particularly those that are already facing a shortage of HCPs [25]. Insufficient numbers of young physicians entering the profession combined with many experienced physicians leaving patient care exacerbate this issue. For instance, in Switzerland, 1 out of every 7 physicians who graduated between 1980 and 2009 eventually opted out of patient care [26]. Moreover, burnout is also a concern among students during medical school and has been found to have a positive correlation with dropout intention [27]. Thus, addressing and mitigating burnout is crucial for the well-being of individuals, the educational system, and the health care system [28].

The impact of digitalization, in particular the introduction of EHR, on clinician well-being has been extensively studied [29-32]. Early EHR implementations were shown to have a negative impact on clinician well-being, reducing job satisfaction and increasing rates of clinician burnout owing to poor system usability, misaligned job roles, and increasing workloads associated with documentation requirements [32,33]. It may be anticipated that technological innovations might have mitigated the situation somewhat; however, at the same time, the pace of technological change has created new challenges such as the need to consider increasing quantities and varieties of data, including patient-reported outcomes [33] and the advances of AI into clinical applications [34]. Previous research suggests an urgent need to prioritize the lived experiences of clinicians when interacting with digital tools to suggest new approaches to design and implement tools to avert negative impacts [35-38].

At present, approaches and interventions aimed at reducing stress and preventing burnout among clinicians primarily involve individual-based practices, including psychoeducation, interpersonal communication, and mindfulness meditation [39]. However, recent findings indicate that enhancing the user experience of health information systems is a crucial factor in reducing stress and improving physician well-being [37,38]. To facilitate improvements in the user experience of EHR systems, strategies have been developed to empower clinicians to collaborate with local administrators, health IT personnel, and EHR developers [35,36]. However, a focus on usability and system design may neglect other important aspects and the effect of digital tools on other human interactions within complex clinical systems [29]. To gain a more comprehensive and mechanistic understanding of the impact of digitalization on clinician well-being, emotions, behaviors, and cognitive processes associated with the use of digital technologies must be explored [40,41]. These questions have largely not been emphasized in previous research [42,43].

### Objective

Previous systematic reviews have explored specific aspects of digital tool integration in health care, offering valuable insights into topics such as mobile health, EHRs, and AI-based technologies [44-46]. These reviews have effectively highlighted the impacts of digital tools on HCP interactions, communication, and documentation, contributing to a better understanding of the advantages of digital tools in health care and their negative impacts on clinician well-being and burnout [15,47-50]. Another review provides comprehensive insights into the positive experiences, facilitators, challenges, barriers, and suggestions for the enhancement of digital care visits [51]. However, most reviews are narrowly focused on specific aspects, overlooking the broader context of health care practices. Moreover, some of these systematic reviews are dated, potentially making their findings less relevant to the current health care landscape as the digital technology evolves. In addition, the frequent lack of firsthand experience from HCPs who use these tools might lead to a limited perspective on their lived experiences.

In this systematic review, we aimed to provide a comprehensive overview of the available evidence on HCPs’ experiences using digital tools in hospital settings. We performed a qualitative synthesis to provide a more nuanced understanding of the impact of digital tools on HCPs’ experiences at work and to offer insights that can inform the development, adoption, implementation, and evaluation of these tools in hospital settings.

## Methods

To investigate the experiences of HCPs using digital tools in clinical settings, we conducted a comprehensive systematic literature review. This review adhered to the updated PRISMA (Preferred Reporting Items for Systematic Reviews and Meta-Analyses) guidelines and was conducted between February and March 2023 (Multimedia Appendix 1) [52].

### Protocol Registration and Amendment

The protocol for this systematic review of qualitative studies has been prospectively registered on PROSPERO (registration number CRD42023393883). We kept the PROSPERO protocol status up-to-date throughout the research process, aligning it with our research’s progress and stages until review completion. No additional modifications were made to the previously published protocol. Before registering the protocol, we conducted PROSPERO searches using various combinations of keywords, including “digital tools,” “healthcare professional,” and “experience” to identify any registered protocols that aim to explore the experience of HCPs with digital tools in hospital settings and to ensure our review makes a significant and novel contribution to this research domain.

### Search Strategy and Information Sources

Our search strategy involved performing a keyword search of peer-reviewed literature published from January 2018 to January 2023 and retrieved from the electronic databases PubMed, Scopus, and Web of Science. The search was limited to the past 5 years to ensure the inclusion of the most current research on the experiences of HCPs, as digital tools evolve over time, and thus, older studies would be less relevant. Our search strategy included keywords such as “digital tools,” “digital applications,” “digital devices,” and “technology” as well as “healthcare professionals” including “clinicians,” “physicians,” and “nurses.” We also used keywords related to “experience” such as “expectation,” “perception,” “adoption,” “acceptance,” and “qualitative.” We used variations of search terms to match synonyms, abbreviations, alternative spellings, and related topics (Multimedia Appendix 2). In addition to the systematic search, we conducted a backward search by reviewing the reference lists of the key publications identified.

### Eligibility Criteria

To be considered for inclusion in the review, the articles had to meet our defined eligibility criteria. We sought to identify qualitative, descriptive interview studies that provided clear and distinct qualitative data and results describing the experiences of HCPs with at least 6 months of experience using digital tools in a hospital setting. Given our primary focus on capturing HCPs’ firsthand experiences with digital tools, we focused our attention on qualitative interview studies. Interviews provide conceptual and theoretical knowledge about people’s life experiences and offer insights into their views, opinions, feelings, knowledge, and expertise [53]. In health-related research, qualitative interviews stand out as a significant approach, allowing individuals to articulate their understanding of the world, leading to deep and novel insights [54]. Unlike other qualitative methods such as ethnography, which observe actions, qualitative interviews allow us to understand the “how” of people’s thinking and lived experiences [55]. Therefore, we also included the qualitative components of mixed methods studies (Multimedia Appendix 3). We defined “experience with digital tools” as the integration of digital tools and technology in health care provisions supporting the achievement of health objectives, including prevention, assessment, diagnosis, consultation, treatment, or monitoring of a patient and medical condition. Our search was limited to peer-reviewed English literature within the defined time frame, population, and setting.

### Selection and Data Collection Process

A panel of 3 independent researchers conducted a rigorous selection process to identify relevant publications for this study. The Covidence web application (Veritas Health Innovation Ltd) [56] was used to screen the titles and abstracts of the studies retrieved from the search strategy by at least 2 reviewers. Any discrepancies were resolved through discussion among the 3 reviewers. Full-text analysis was then performed by 2 authors to assess eligibility, with clear reasons provided for exclusion, and any disagreements were resolved by the third author.

To ensure accurate and consistent data extraction and quality assessment, we developed templates for recording study characteristics, including general publication information, key study and method characteristics, study population and background characteristics, and key findings. We used the “Critical Appraisal Skills Program” qualitative assessment checklist (Multimedia Appendix 4) to evaluate the methodological quality and validity of the study results. Data were independently collected and assessed by 2 authors, and any disagreements were resolved through discussion with the third author.

### Data Items and Synthesis

For data analysis and management, “ATLAS.ti” software (Scientific Software Development) [57] was used to allow line-by-line coding by 2 reviewers to capture key data and identify recurrent topics. Primary codes were then compared and synthesized to derive descriptive themes and higher-order constructs based on grouping, reviewing, and analyzing similar topics and concepts in the primary codes underlying the experiences of HCPs using digital tools in a hospital setting. To ensure a comprehensive approach, we used iterative coding and synthesis of codes, considering the findings from a thorough review of the theoretical frameworks presented in the existing literature. This iterative process supported the development of a novel theoretical framework specific to this study. The framework was then continuously evaluated through its application to the coding process, allowing for refinements and adjustments as necessary.

## Results

### Study Selection

**Figure 1:**
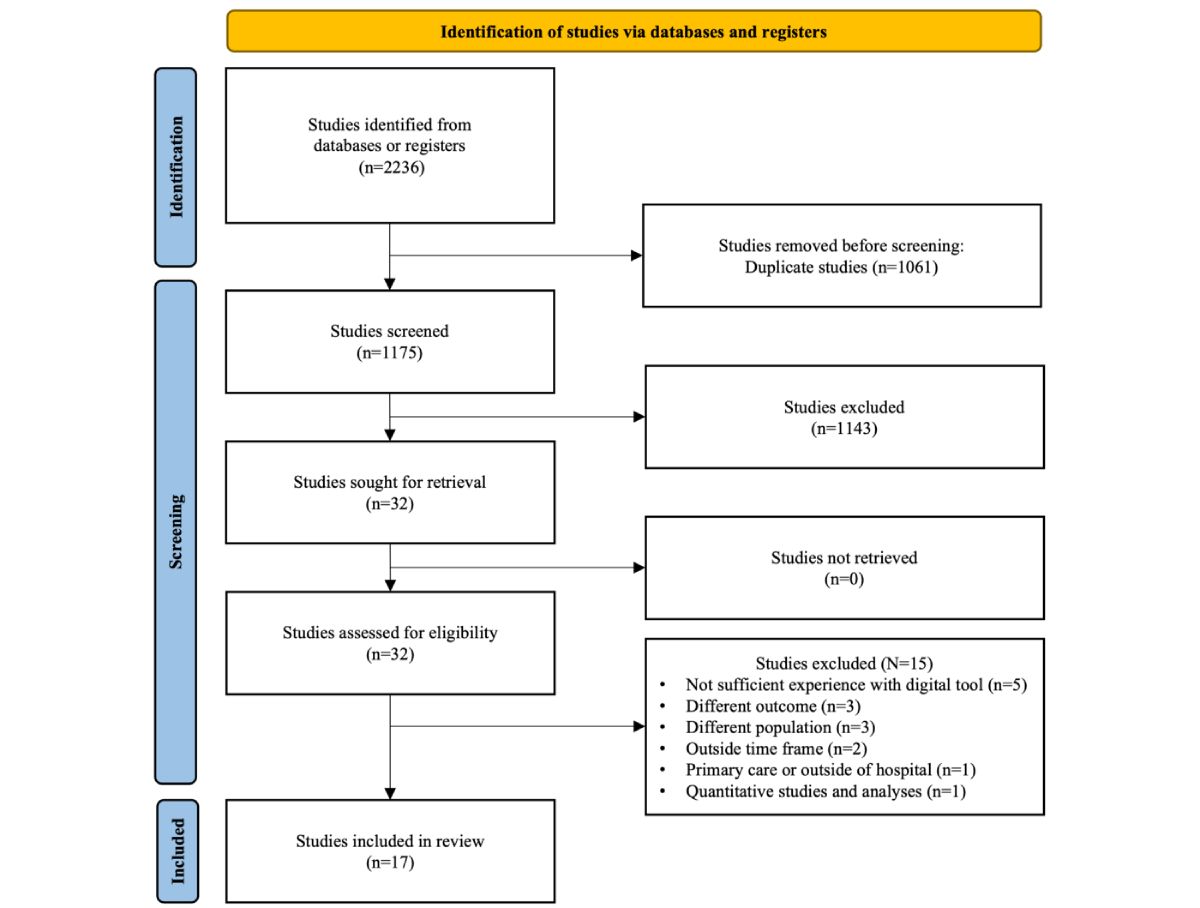
Flow diagram of the study selection process.

In total, 2236 publications were identified, of which 1061 (47.45%) were removed owing to duplication. Subsequently, during the initial screening phase, 1143 (51.12%) articles were excluded based on predefined inclusion and exclusion criteria. The remaining 32 (1.43%) studies underwent a thorough full-text review, leading to the further exclusion of 15 (0.67%) articles owing to insufficient experience of HCPs with the respective digital tools (n=5, 33%), outcomes that focused on factors other than the experience of HCPs (n=3, 20%), excluded study populations (n=3, 20%), publication date outside the time frame (n=2, 13%), exclusion of study location and setting (n=1, 7%), and quantitative study analysis (n=1, 7%). Ultimately, 17 studies were included in the review (Figure 1).

### Study Characteristics

**Figure 2:**
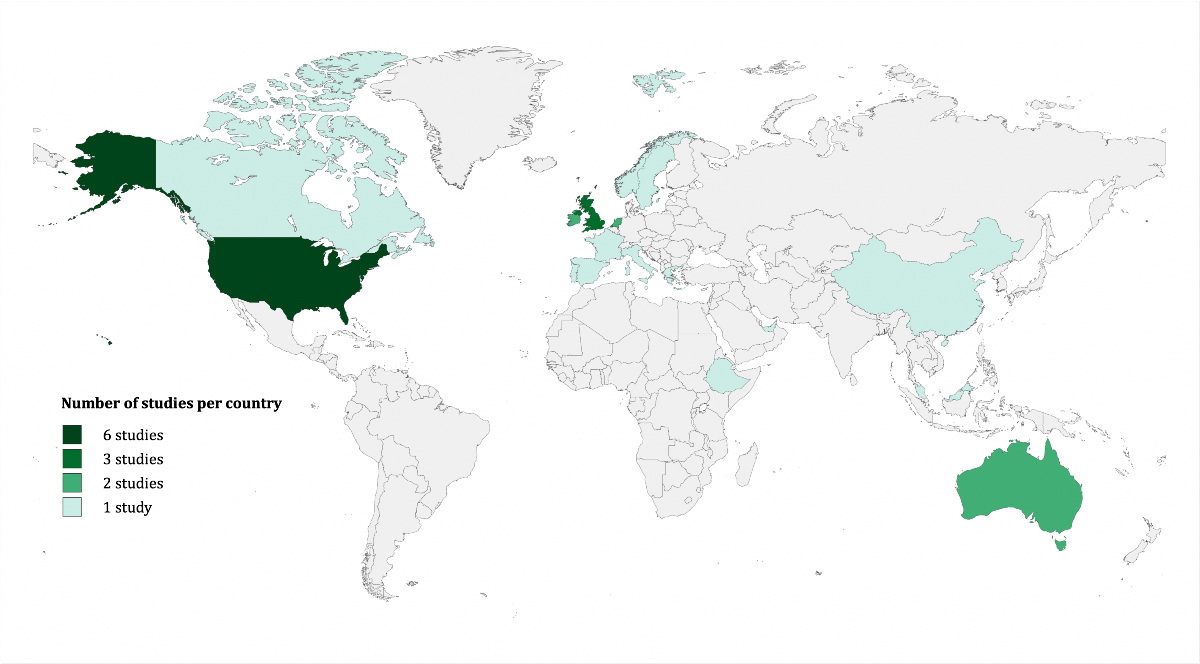
Geographic distribution of studies by country.

All 17 selected publications focused on HCPs’ experiences of using digital tools in their day-to-day practice. Of the 17 studies, 5 (29%) focused exclusively on physicians [58-62], 4 (24%) focused solely on nurses [63-66], and 8 (47%) had a mixed population of clinicians and associated staff of the health care team [66-74]. More than half (10/17,59%) of the studies reported on CDS tools, with 29% (5/17) of the studies investigating conventional CDS [62,63,66,67,71] and 29% (5/17) of the studies focusing on AI-based CDS [59,61,65,69,70]. Of the 17 studies, 6 (35%) focused on EHRs [58,60,64,68,73,74], whereas the remaining 1 (6%) study examined a remote patient-monitoring tool [72]. Of the 17 studies, 13 (76%) were solely based on qualitative individual semistructured interviews. Of the remaining 4 studies, 2 (50%) adopted a combination of qualitative techniques, consisting of individual semistructured interviews, focus group interviews, field notes, and direct observation. Of the 17 studies, the other 2 (12%) followed a mixed methods approach [61,64]. They conducted qualitative individual semistructured interviews and enriched their data with quantitative surveys using the 5-point Likert scale [66,74]. The studies were conducted in 26 different locations, with 6 (23%) studies conducted in the United States [58,59,62,65,68,70], 3 (12%) in the United Kingdom [67,68,71], 2 (8%) in Ireland [68,71], 2 (8%) in the Netherlands [69,72], and 2 (8%) in Australia [64,68]. Furthermore, single studies were conducted in Europe, including Norway [73], Sweden [63], France [71], Italy [71], Spain [71], and Portugal [71]; Canada [60]; Asia, including the United Arab Emirates [68], China [66], and Malaysia [61]; and Ethiopia in Eastern Africa [74] (1/26, 4%; Table 1; Figure 2).

**Table 1:** Overview of the publications selected for analysis.

Study	Publication title	Location	Study aim	Findings
Asan et al [58]	Oncologists’ views regarding the role of electronic health records in care coordination	United States	Assessment of oncology providers’ perceptions of EHRs^a^ for supporting communication with patients and coordination of care with other providers	EHRs did not adequately support the teamwork of oncology providers, which could lead to potential hazards in the care of oncological patients.
Carlisle et al [67]	Clinicians’ experiences of using and implementing a medical mobile phone app (QUiPP^b^ V2) designed to predict the risk of preterm birth and aid clinical decision making	United Kingdom	Exploration of clinicians’ experiences of using and implementing the QUiPP app (clinical decision-making individualizing risks of early delivery within the relevant time frame) in clinical practice	The organizational and cultural context at different sites appeared to have a large impact on app implementation and the experience of physicians.
Choudhury et al [59]	Clinicians’ perceptions of an artificial intelligence-based blood utilization calculator: qualitative exploratory study	United States	Investigation on how clinicians perceived this AI^c^-based decision support system and, consequently, understand the factors hindering BUC^d^ use	Analytical efficacy alone does not guarantee technology adoption; it relies on the system’s design, user perception, and knowledge. AI systems should be self-explanatory in their use instructions, and using technology outside its intended audience limits user perception and use.
Cronin et al [68]	A qualitative analysis of the needs and experiences of hospital-based clinicians when accessing medical imaging	Ireland, United Kingdom, United Arab Emirates, United States, and Australia	Exploration of health care professionals’ experiences, practices, and preferences when using PACS^e^ to identify shortcomings in the existing technology and inform future developments	Health care professionals rely on the PACS in their workflow, but there is a lack of awareness and limited use of its advanced features. Training; enhanced usability; and the adoption of touchless, voice-controlled PACS are viewed positively by most users and would bring benefits.
Drogt et al [69]	Integrating artificial intelligence in pathology: a qualitative interview study of users’ experiences and expectations	Netherlands	Investigation of the integration of AI within pathology through in-depth interview to gain insight into the professional stance toward possibilities for AI integration and to analyze the connection to the broader social and ethical context of AI development while focusing primarily on the issue of responsibility	Pathologists generally support the integration of AI owing to its potential benefits but emphasize the importance of cautious implementation. Three key recommendations for AI integration include maintaining a pragmatic approach, providing task-specific information and training, and allowing time for reflection on evolving roles and responsibilities.
Fishbein et al [60]	Physician experience with electronic order sets	Canada	Exploration of physicians’ perspectives and experiences using electronic order sets	System usability depends on factors such as ease of use, workflow improvement, and simple design, but searchability issues can complicate navigation. Electronic order sets enhance patient safety by reducing reliance on physician memory, providing real-time access to best practices, and enabling individualized care.
Henry et al [70]	Human-machine teaming is key to AI adoption: clinicians‚ experiences with a deployed machine learning system	United States	Understanding the role that clinicians see machine learning as playing in acute clinical care and pathways and barriers to building trust with machine learning–based recommendation	Collaboration with a machine learning system is facilitated by viewing it as a supportive validation tool across workflows, building trust through experience. However, concerns include overreliance and potential harm from standardized care, emphasizing the need for clinicians to be willing and able to integrate system information into patient care.
Holmström et al [63]	Registered nurses’ experiences of using a clinical decision support system for triage of emergency calls: a qualitative interview study	Sweden	Description of how registered nurses make use of a CDSS^f^ to triage calls to emergency medical dispatch centers, from the perspective of professional autonomy	CDSSs can enhance the autonomy of nurses in patient assessments, but further improvements are needed in areas such as technical optimization, interoperability, and nurse education and training on the system.
Jacob et al [71]	Clinicians’ role in the adoption of an oncology decision support app in Europe and its implications for organizational practices: qualitative case study	United Kingdom, Ireland, France, Italy, Spain, and Portugal	Understanding clinicians’ roles in the adoption of an oncology decision support app, the factors impacting this adoption, and its implications for organizational and social practices	Clinicians’ adoption of the decision support app was influenced by app-specific features, social factors, and internal organizational dynamics. The app facilitated workflow efficiency, improved practice, and offered location flexibility, but adoption was hindered when cultural acceptance was lacking or interoperability with other digital systems was limited.
Jedwab et al [64]	Nurses’ experiences after implementation of an organization-wide electronic medical record: qualitative descriptive study	Australia	Exploration of Australian nurses’ postimplementation experiences of an organization-wide EHR system	Implementing an EMR^g^ impacted nurses’ autonomy, workflow, and professional role, with motivation identified as a crucial factor in adapting to the new system. When implementing a new system, considering motivation becomes essential to ensure successful adoption.
Jongsma et al [72]	How digital health affects the patient-physician relationship: an empirical-ethics study into the perspectives and experiences in obstetric care	Netherlands	Exploration of the perspectives of patients and health care providers on the patient-physician relationship in digital health, focusing on roles and responsibilities in perinatal care and the influence of technology on medical decision-making	Digital health had both positive and negative impacts on the patient-physician relationship, enabling patients to access their health data but causing confusion regarding when to alert a physician. The study led to 6 ethical recommendations based on shared responsibility for measurements.
Jordan et al [65]	The impact of cultural embeddedness on the implementation of an artificial intelligence program at triage: a qualitative study	United States	Exploration of the cultural and technological elements of the implementation of an AI CDS^h^ aid in an emergency nursing triage process in an urban community hospital	Initially met with skepticism, the AI program eventually supported triage decision-making for emergency nurses but could not assist with culturally nuanced decisions. Sufficient resources and workforce were crucial for technology acceptance.
Kalayou et al [74]	Physicians’ attitude towards electronic medical record systems: an input for future implementers	Ethiopia	Analysis of physicians’ attitudes regarding EMRs and the predictive factors that may influence their attitudes. As a result, the findings will have an influence on future adoption success and physician acceptability of EMR systems	The implementation of EMR was directly linked with ownership of own digital hardware and health care professionals valued it for the digital availability of patient data. Lack of training and experience on EMR systems was a hindering factor.
Olakotan and Yusof [61]	Evaluating the appropriateness of clinical decision support alerts: a case study	Malaysia	Evaluation of the appropriateness of CDS alerts in supporting clinical workflow from a sociotechnical perspective	Workflow success depends on factors beyond CDS design and features, including sociotechnical elements, organizational processes, and work dynamics. Although well-designed CDS is valuable, it cannot substitute for medical skills, knowledge, and adequate training.
Richardson et al [62]	Barriers to the use of clinical decision support for the evaluation of pulmonary embolism: qualitative interview study	United States	Exploration of the psychological and behavioral barriers to the use of a CDS tool	Psychological and behavioral barriers, such as fear of missing a pulmonary embolism and time pressure, hindered the use of CDS. Support from hospital leadership, adequate training, and trust can promote CDS adoption.
Smaradottir and Fensli [73]	User experiences and satisfaction with an electronic health record system	Norway	Analysis of the user experiences, perceived usability, and the attitudes among health care professionals toward a specific EHR system that is commonly used	Limited familiarity with the EHR system led to underuse of features. Challenges with interoperability and patient data storage compromised safety, whereas patient involvement as a third-party user remains unaddressed.
Zhai et al [66]	Transition to a new nursing information system embedded with clinical decision support: a mixed-method study using the HOT^i^-fit framework	China	Investigation of nurses’ perceptions and experiences with transition to a new nursing information system 2 y after its first introduction	Successful implementation of a new nursing information system required collaboration between end users, administrators, and technical personnel. Nurses should be involved in system development to optimize user experience and system usability.

^a^EHR: electronic health record.

^b^QUiPP: quantitative innovation in predicting preterm birth.

^c^AI: artificial intelligence.

^d^BUC: blood utilization calculator.

^e^PACS: Picture Archiving and Communications Systems.

^f^CDSS: Clinical Decision Support System.

^g^EMR: electronic medical record.

^h^CDS: clinical decision support.

^i^HOT: human, organization, and technology.

### Theoretical Framework

**Figure 3:**
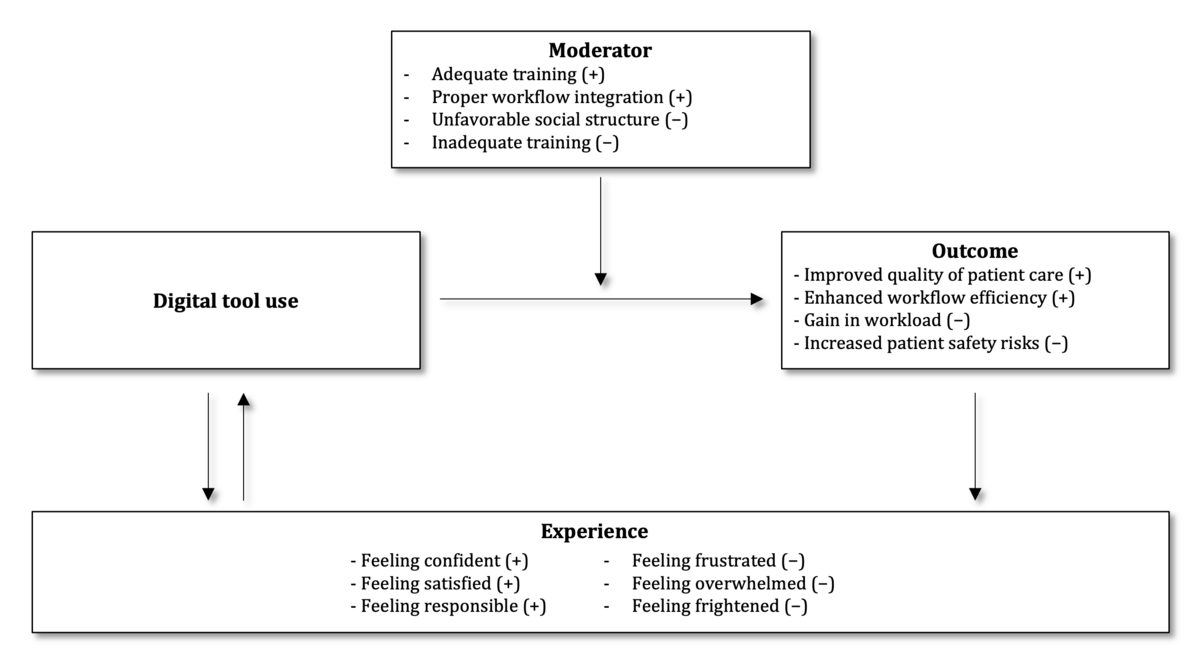
Proposed theoretical framework distinguishing moderators; outcomes; and experience of using digital tools, including positive and negative examples.

Our preliminary assessment of the literature highlighted the need for a theoretical framework to understand the complex interplay between the use of digital tools, experience, and outcomes within clinical and general workflows. In recent years, several theoretical frameworks have been developed to predict and explain the acceptance behavior of new technologies [75]. In the health care context, the Technology Acceptance Model and the Unified Theory of Technology Acceptance and Use are among the most widely used models for predicting acceptance behavior [76]. However, direct experiences when using tools, which are potential moderators for the downstream impact on well-being, are often not distinguished from other outcomes or moderators. Building on this literature and informed by our thematic analysis of the included studies, we defined a theoretical framework to distinguish and illustrate connections between using digital tools, the experience of using digital tools, moderators that seem to impact the use of digital tools positively or negatively, and outcomes as a result of using the tools (Figure 3).

The use of digital tools, such as EHRs and CDS, is typically aimed at achieving specific goals such as improving patient care, enhancing workflow efficiency, and increasing information availability, all of which are potential outcomes of digital tool use. The positive outcomes of using digital tools include improved quality of patient care, enhanced workflow efficiency, and better information availability. Negative outcomes can include increased workload, increased patient safety risks, and disruptions in the workflow.

Certain moderators can have positive or negative effects on digital tool use. Examples of positive moderators include adequate training, proper workflow integration, and a user-friendly interface design, whereas negative moderators can include unfavorable social structures, inadequate training, and insufficient interface design and customization.

The framework explicitly includes the experiences of each individual user as a separate construct. Experiences are private to the individual, encompassing thoughts, emotions, and feelings. They can be influenced by either the outcome of using digital tools or using the tool itself, which plays a crucial role in further promoting or hindering the use of digital tools either positively or negatively. Thus, as indicated in Figure 3, there are possibilities for the development of positive or negative feedback cycles.

In the subsequent sections, we present our findings using this theoretical framework and provide a comprehensive analysis of the relationships between digital tool use, moderators, experience, and overall outcomes.

### Frequency of Reported Themes

#### Overview

Our analysis and synthesis of themes resulted in the identification of 6 overall constructs according to our theoretical framework, encompassing positive and negative experiences of HCPs when using digital tools, positive and negative moderators that possibly affect their adoption and use, and the corresponding positive and negative effects and outcomes of the use of digital tools may result in (Table 2). Overall, clinician experiences were less frequently reported as compared with moderators or outcomes, with positive experiences reported in 31 annotations and negative experiences reported in 40 annotations. Overall, moderators were the most frequently reported phenomena across publications, with 194 annotations on positive moderators and 121 annotations on negative moderators. Furthermore, 108 positive and 131 negative annotations for outcomes were identified (Multimedia Appendix 5).

**Table 2:** Most frequently emerging themes and topics in the selected studies (N=17).

Category and most frequently reported topics			Publications, n (%)		Exemplary quote from study
**Positive experience**					
	Feeling confident	10 (59)		“Nurses attributed reinforcement of their triage process to AI^a^ feedback, which increased their confidence.” [65]	
	Feeling responsible	6 (35)		“However, they saw themselves as maintaining ultimate responsibility for diagnosis and treatment decisions.” [70]	
	Expressing satisfaction with the tool or situation	6 (35)		“The work here with emergency triage builds on my experience in emergency nursing to a great extent.” [63]	
	Feeling grateful	4 (24)		“[Clinicians] acknowledged the benefits of having the BUC [blood utilization calculator]...” [59]	
**Negative experience**					
	Feeling frustrated	8 (47)		“The lack of online access to scans performed in some hospitals is a clear source of frustration for certain HPs^b^...” [68]	
	Feeling overwhelmed by information load	7 (41)		“Providers also reported that they may be overwhelmed by the number of in-basket messages...” [58]	
	Feeling frightened	7 (41)		“Nurses’ anxiety about needing to learn and use a new system, stress related to additional pressures in an already busy work environment, and fear and resistance to change with the EMR^c^ implementation emerged as emotional barriers to EMR use by nurses.” [64]	
	Feeling confused	5 (29)		“...while others perceived it to be confusing and hard to use, since the technology was not tailored to their needs.” [59]	
**Positive moderator**					
	Sufficient training	11 (65)		“...physicians who got EMR training had more knowledge about the system than their colleagues, which improved their attitude and motivation towards the system.” [74]	
	Adequate workflow integration	10 (59)		“The EMR implementation was described as successful by nurses when they felt that they had learned the system and adapted their ways of working and workflows.” [64]	
	Favorable organizational structures	8 (47)		“...there are other social and organizational factors that play a crucial role in the adoption and success of such new technologies...” [71]	
	User-friendly design of interface	7 (41)		“User-centered design, wherein the user is centrally involved in all phases of the design process, is essential for AI health care technologies.” [59]	
**Negative moderator**					
	Unfavorable social structures	9 (53)		“...there are also social and organizational aspects such as shortage of time and financial resources that can cause limitations to such solutions’ adoption.” [71]	
	Lack of training	8 (47)		“Lack of continuity of training was also a problem for nurses.” [66]	
	Lack of a tailored tool design	6 (35)		“...others perceived it to be confusing and hard to use, since the technology was not tailored to their needs.” [59]	
	Insufficient design of user interface	6 (35)		“Also, poorly designed alert interfaces have led to difficulty in retrieving patient information, which may lead to cognitively based errors and impedes the performance of clinicians.” [61]	
**Positive outcome**					
	Improvement in quality of patient care	12 (71)		“The system has improved care quality by reducing medication errors.” [61]	
	Increase in workflow efficiency	10 (59)		“HPs report that the introduction of PACS^d^ had a dramatic impact on the clinicians’ working day, bringing a newfound convenience to the clinical workflow.” [68]	
	Better information availability	8 (47)		“Specifically, PACS has increased the amount of useful information available to clinicians, and improved the availability of images...” [68]	
	Increase in patient safety	6 (35)		“...the use of order sets increased safety by ensuring that physicians followed evidence-based practices and minimized the possibility of omitting important interventions.” [60]	
**Negative outcome**					
	Increase in workload	13 (76)		“Clinicians often had to figure out a way to bypass the system and place their blood transfusion order, adding to their existing workloads and slowing down the transfusion process.” [59]	
	Increased patient safety risks	8 (47)		“The loss of nurses’ narrative owing to EMR use was raised as a concern for patient safety...” [64]	
	Missing or outdated information	8 (47)		“Another user problem is the copy and paste of text between sections in the record, which might produce potentially outdated and inaccurate information.” [73]	
	Complications and interruption in workflow	7 (41)		“Overriding default options before completing prescriptions has increased workflow disruption in dermatological settings.” [61]	

^a^AI: artificial intelligence.

^b^HP: health professional.

^c^EMR: electronic medical record.

^d^PACS: Picture Archiving and Communications Systems.

#### Positive Experiences of HCPs Using Digital Tools

Almost all studies reported positive experiences of HCPs using digital tools (Multimedia Appendix 5). The most frequently reported experiences were feeling confident about using a tool (10/17, 59%), feeling responsible (6/17, 35%), being satisfied with a tool or situation (6/17, 35%), and feeling grateful (4/17, 24%). Other experiences that were less frequently reported include feeling comfortable using the tool; expressing appreciation; feeling autonomous and empowered; and feeling supported, encouraged, or optimistic.

#### Negative Experiences of HCPs Using Digital Tools

Of the 17 studies analyzed, 14 (82%) reported negative experiences of HCPs using digital tools (Multimedia Appendix 5). The most frequently reported negative experiences were frustration (8/17, 47%) owing to various reasons, such as communication issues, deteriorated physician-patient interaction, lack of sufficient resources, increased workload, difficulties in adapting to an unintuitive system, challenges in finding information within the EHR system, and limited or impaired access to web-based information stored within digital systems. Other commonly reported negative experiences were feeling overwhelmed by information (7/17, 41%) and various fears (7/17, 41%), including fear of change and replacement, fear of forgetting, or fear of losing or misinterpreting information. Moreover, feeling confused was mentioned owing to a conflict with the professional identity of HCP. This conflict stemmed from the impact of digital tools on their perceptions of their professional image, concerns about their work visibility, as well as their perception of digital tools as a threat to their professional autonomy (5/17, 29%). Other negative experiences that were less frequently reported included feeling disrupted, feeling concerned mainly for the patient, feeling disappointed by the tool, feeling uncertain, feeling unsatisfied with work situations, feeling stressed, or even feeling shocked.

#### Moderators With a Potential to Positively Influence Digital Tool Use

We identified several moderators that possibly result in positively impacting HCPs’ use of digital tools, such as sufficient tool design, improved patient care and safety, and favorable structural factors. The most reported factors that reinforced the use of digital tools were sufficient training (11/17, 65%), workflow integration (10/17, 59%), favorable organizational structures (8/17, 47%), and well-designed user interfaces (7/17, 41%). Other relevant factors include the HCPs’ perception that the tool supports clinical excellence, quick and easy information access, trust in the tool, an appropriate workstation setup, and a great extent of prior use or familiarity with the tool or technologies.

#### Moderators With a Potential to Negatively Influence Digital Tool Use

Conversely, negative moderators have been reported that potentially hinder or limit the use of digital tools. We identified various moderators that may have a negative impact on HCPs’ use of digital tools, such as technical issues and a nonintuitive interface design, unfavorable structures, personal attitude, limited prior exposure, and concerns about patient care and data privacy. Unfavorable social and organizational structures (9/17, 53%), the lack of training (8/17, 47%), insufficient user interface design (6/17, 35%), and the lack of tailored tool design and features (6/17, 35%) were the most frequently reported negative moderators. Other negative moderators include time constraints, insufficient workstation setup, the lack of workflow integration, and limited or impaired information accessibility.

#### Positive Effects and Outcomes of Digital Tool Use

Studies reported several positive outcomes resulting from the use of digital tools. These included patient-centered care and empowerment, improved quality of care, streamlined workflow and productivity, efficient information management, optimized cognitive support of HCPs, and collaborative care. The most frequently reported positive outcomes were improved quality of patient care (12/17, 71%), increased workflow efficiency (10/17, 59%), better information availability (8/17, 47%), and increased patient safety (6/17, 35%). Other frequently reported positive outcomes included improved time efficiency through quick and easy access to information, the promotion of critical thinking, and a reduction in errors.

#### Negative Effects and Outcomes of Digital Tool Use

The use of digital tools also resulted in negative outcomes. These included communication and information management challenges, issues with information accuracy and availability, patient safety risks, reduced quality of care, and organizational and workflow issues. The most frequently reported negative outcomes were increased workload (13/17, 76%), patient safety risks (8/17, 47%), missing or outdated information (8/17, 47%), and complications or interruptions in the workflow (7/17, 41%). Other reported negative outcomes included time-consuming information management, incomplete information transfer, inefficiencies in the documentation process, and reduced or suboptimal patient care overall.

### Differences in the Themes Reported by the Types of Tool

Of the 17 identified studies, most focused on CDS systems, including 5 (29%) on conventional CDS systems [62,63,66,67,71] and 5 (29%) on AI-based CDS systems [59,61,65,69,70]. Moreover, 6 (35%) out of 17 studies focused on EHR systems [58,60,64,68,73,74], and 1 (6%) study focused on a remote patient-monitoring device [72], which did not fit into any of the 3 broader categories (Table 3).

Across all digital systems, gain in confidence was the most frequently reported positive experience for users (conventional CDS [66,67], AI-based CDS [61,65], and EHR [64,68,73,74]). Furthermore, feeling satisfied was reported for EHR [58,68,73] and conventional CDS systems [63,67] but not for AI-based CDS systems. However, clinicians expressed gratitude [59,70], encouragement [59], hopefulness [69], and feeling supported [65] when using AI-based tools, which was not observed for the other systems.

The most reported negative experience for conventional CDS systems was feeling disrupted [62,66]. In contrast, for AI-based CDS tools, the most frequently cited negative experience was feeling frightened [65,69]. Although frustration was the most frequently mentioned negative experience in EHR systems [58,60,64,68,73,74], only a few publications mentioned it for conventional [66] and AI-based [65] CDS systems. The same also applied to feeling overwhelmed by information [58,60,68,73]. Similarly, feeling insecure, shocked, stressed, and unsatisfied with the work situation [64] was only mentioned for EHRs and not for the CDS tools. In contrast, uncertainty was only reported for conventional [69] and AI-based [67] CDS systems but not for EHRs.

The primary moderators that may positively impact the use of digital tools were largely consistent across all electronic systems. Sufficient training was deemed highly important for conventional CDS [62,66,71], AI-based CDS [59,61,69,70], and EHR [64,68,73,74] systems. Similarly, sufficient workflow integration was mentioned for conventional CDS [62,67,71,74], AI-based CDS [61,69], and EHR [58,64,68,74] systems. For AI-based CDS tools, trust [59,69,70] and the perception of support [59,69,70] were reported as highly critical factors to enhance use. Moreover, it is essential for AI-based CDS tools to provide clinicians with a sense of advice and collaboration, augmenting their choices and assisting in their day-to-day work. In the case of CDS AI-based tools, creating a perception of being an adviser and cooperating partner, along with a deep understanding of the fundamental aspects of the tool [69,70], was found to be of significant importance when compared with other tools. In contrast, for EHRs, favorable organizational structures [60,64,68,74] and providing quick and easy access to information [58,60,68] were reported as essential for using the system. Furthermore, the fear of negative consequences [64], sufficient IT infrastructure [60], commoditization of the tool [68], and the perception of a service to the community [68] were only mentioned for EHR systems.

Across all studies, HCPs commonly reported unfavorable organizational structures as the most critical negative moderator for the use of conventional CDS [66,67,71], AI-based CDS [61,65], and EHR [60,64,68,74] systems. In addition, unfavorable social pressure was mentioned for conventional CDS tools [62,67,71]. In addition, the lack of training was identified as a negative factor, particularly for EHRs [64,68,73,74] but also for conventional CDS [66,67] and CDS AI-based [61,65] systems. In addition, for EHRs only, insufficient user interface design [11,14,19,20], workstation setup [58,68,73,74], and data privacy concerns were mentioned [64,68]. In contrast, for AI-based CDS systems, the lack of tailored design [59,69] and distrust [65,70] were reported as negative moderators. In addition, unfavorable features for AI-based CDS [65] and conventional CDS [63] systems, high costs (AI-based CDS [69] and conventional CDS [71]), and negative attitudes toward technology (AI-based CDS [69] and conventional CDS [71]) were only reported for CDS systems but not for EHR.

In terms of positive outcomes, all studies focusing on EHR mentioned better information availability [58,60,64,68,73,74] as the major result of using EHR in hospitals. In addition, improvements in the quality of patient care were reported across all tools, including conventional CDS [62,66,67,71], AI-based CDS [61,65], and EHR [58,60,64,68,74] systems. Workflow efficiency was also found to increase with the use of conventional CDS [61,67,71], AI-based CDS [61,69], and EHR [58,60,68,73,74] systems. Furthermore, all tools reported an increase in patient safety (conventional CDS [62,77], AI-based CDS [61,65], and EHR [60,74]) and a gain in time efficiency (conventional CDS [67,71], AI-based CDS [69,70], and EHR [60,68]). Moreover, it was reported that AI-based CDS tools, in particular, foster critical thinking [59,65,70], whereas conventional CDS tools were specifically associated with a better patient experience [67,71]. In contrast, it was reported that EHRs offered quick and easy access to information [58,60,64,68], and this was the only tool type for which better documentation [64] and cost savings [68] were reported.

**Table 3:** Most emerging themes and topics per tool.

Tools and category		Most emerging theme (number of publications)
**Conventional CDS^a^ (n=5)**		
	Positive experience	Feeling confident (n=2) Feeling satisfied (n=2)
	Negative experience	Feeling disrupted (n=2)
	Positive moderator	Sufficient workflow integration (n=5) Sufficient training (n=4)
	Negative moderator	Unfavorable organizational structure (n=3) Unfavorable social pressure (n=3)
	Positive outcome	Improved quality of patient care (n=4) Increased workflow efficiency (n=3)
	Negative outcome	Information missing or outdated (n=4) Workload gain (n=3)
**AI^b^-based CDS (n=5)**		
	Positive experience	Feeling confident (n=2) Feeling grateful (n=2) Feeling responsible (n=2)
	Negative experience	Feeling frightened (n=2)
	Positive moderator	Sufficient training (n=4) Perception of support (n=3) Trust in tool (n=3)
	Negative moderator	Unfavorable organizational structure (n=2) Lack of training (n=2) Lack of tailored design (n=2) Distrust (n=2)
	Positive outcome	Fostering critical thinking (n=3)
	Negative outcome	Workload gain (n=4) Patient care suboptimal (n=3)
**EHR^c^ (n=6)**		
	Positive experience	Feeling confident (n=4) Feeling satisfied (n=3)
	Negative experience	Feeling frustrated (n=5) Feeling overwhelmed (n=4)
	Positive moderator	Sufficient workflow integration (n=4) Sufficient training (n=4) Favorable organizational structure (n=4)
	Negative moderator	Unfavorable organizational structure (n=4) Lack of training (n=4) Insufficient workstation setup (n=4) User interface design insufficient (n=4)
	Positive outcome	Better information availability (n=6) Improved quality of patient care (n=5) Increased workflow efficiency (n=5)
	Negative outcome	Workload gain (n=5)

^a^CDS: clinical decision support.

^b^AI: artificial intelligence.

^c^EHR: electronic health record.

The most frequently reported negative outcome across all tools was an increase in workload (conventional CDS [62,66,67], AI-based CDS [59,61,65,69], and EHR [58,64,68,73,74]). In addition, missing and outdated information was often reported for EHR [58,64,73,74] and conventional CDS [62,63,66,77] systems. For AI-based CDS tools, reduced quality of patient care [61,70,71], patient harm [59,70], and increased patient safety risks [59,61] were reported, which were also mentioned for EHR systems [58,64,68,73]. Lack of tool objectivity was only reported for CDS systems (conventional CDS [63] and AI-based CDS [69]). In contrast, time-consuming information management [58,64,73,74] and workflow complications or interruptions [64,68,73,74] were reported twice as much for the use of EHR than for CDS systems. Furthermore, information overload [58,64], increase in human errors [58,68], incorrect information transfer [58,68], and reduced face-to-face collaboration time for physicians [64,68] were also solely reported for the use of EHR systems.

### Differences in Themes Reported by Population

Of the 17 identified publications, 8 (47%) focused on mixed populations of HCPs [67-74], 5 (29%) explored the experiences of physicians only [58-62], and 4 (24%) investigated the experiences of nurses [63-66] (Table 4).

The analysis of the experiences of physicians and nurses as individual population groups revealed that nurses more frequently reported feeling confident and supported by health care tools [64-66] as compared with physicians [61]. However, both nurses and physicians reported feeling satisfied, responsible, and grateful [58-60,63,64] with the tools. Furthermore, physicians expressed feeling comfortable and encouraged [58,59], whereas nurses did not report such feelings.

In terms of negative experiences, physicians commonly expressed feeling overwhelmed by information [58,60,61], confused [59,62], and disrupted [61,62]. In contrast, nurses more frequently reported feeling frustrated [64-66], frightened [64,65], and concerned [64,65].

Both physicians and nurses identified sufficient workflow integration (physicians [58,61,62] and nurses [64,66]) and adequate training (physicians [59,62,72] and nurses [64,66]) as the most important positive moderators. In addition, physicians considered adequate user interface design [59-61] to be highly significant, whereas nurses identified cultural flexibility [65,66] as an essential factor.

Negative moderators with the potential to hinder the use of digital tools were identified by both nurses and physicians. Nurses mostly reported a lack of training [64-66], whereas physicians commonly reported a lack of workflow integration [58,61,62] as the main challenge. In addition, both groups of HCPs identified unfavorable organizational structure (physicians [60,61] and nurses [64,66]) and insufficient user interface design (physicians [58,61] and nurses [64,66]) as negative moderators that can impede the use of digital tools. Moreover, physicians were more likely than nurses to report a lack of workstation setup as a hindrance [58,61].

In terms of positive outcomes, both physicians and nurses reported an improvement in patient care quality (physicians [58,60-62] and nurses [64-66]) with digital tools. Nurses highlighted the reduction of errors, whereas physicians emphasized better information availability [58,60,61], increased workflow efficiency [58,60,61], and improved patient safety [60-62]. Both groups acknowledged the importance of cognitive support and fostering critical thinking (physicians [59,60] and nurses [63,64]). Physicians reported better adherence to guidelines [59,60] and information transfer [58,61], whereas nurses valued better prioritization and documentation [64].

However, the use of digital tools also had negative outcomes for both groups. Workload gain was the most commonly reported negative outcome (physicians [58,59,61,62] and nurses [64-66]), followed by patient safety risks (physicians [58,59,61,62] and nurses [64,65]) and time-consuming information management (physicians [58,61,62] and nurses [64,66]). Physicians specifically mentioned incomplete information transfer [58,61,62], whereas nurses cited missing or outdated information and inefficiencies in the documentation process [63,64,66] as additional negative outcomes of using digital tools. Moreover, physicians reported concrete patient harm [58,59] and a lack of addressing psychological and emotional issues of patients [58,62] as negative outcomes.

**Table 4:** Most emerging themes and topics per study population.

Population and category		Most emerging theme (number of publications)
**Physicians (n=5)**		
	Positive experience	Feeling confident (n=1) Feeling responsible (n=1) Feeling satisfied (n=1)
	Negative experience	Feeling overwhelmed (n=3) Feeling confused (n=2) Feeling disrupted (n=2)
	Positive moderator	Sufficient workflow integration (n=3) Sufficient training (n=3) Sufficient user interface design (n=3)
	Negative moderator	Lack of workflow integration (n=3)
	Positive outcome	Improved quality of patient care (n=4)
	Negative outcome	Workload gain (n=4) Patient safety risk (n=4)
**Nurses (n=4)**		
	Positive experience	Feeling confident (n=3)
	Negative experience	Feeling frustrated (n=3) Feeling frightened (n=2) Feeling concerned (n=2)
	Positive moderator	Sufficient training (n=2) Sufficient workflow (n=2) integration Cultural embeddedness (n=2)
	Negative moderator	Lack of training (n=3)
	Positive outcome	Improved quality of patient care (n=3) Better information availability (n=3)
	Negative outcome	Workload gain (n=3) Information missing or outdated (n=3) Inefficiencies in documentation process (n=3)

## Discussion

### Principal Findings and Significance

Digital transformation is altering many aspects of the health care system and the accompanying clinical workflows. Many of these changes are improvements with the potential for more and easier access to information and innovations in workflows toward better care; however, there are also concerns about possible unintended consequences. The interactions between clinicians and digital tools and systems are the direct frontier of digital transformation, affecting clinical work, roles, team dynamics, and clinical encounters with patients. As mentioned in the *Introduction* section, previous studies have extensively explored the impact of digitalization, particularly the introduction of EHR, on clinician well-being. Early findings indicated that EHR implementations had negative effects, leading to reduced job satisfaction and increased rates of clinician burnout. Our systematic literature review aimed to provide an up-to-date overview of the literature encompassing the perspective of clinicians using digital tools in hospital settings.

Our first finding was that despite the many calls to take clinician experiences into consideration, the body of research addressing this topic is still quite small, and only 17 studies since 2018 met all inclusion criteria. We found that many of the studies retrieved by the search but subsequently discarded were explorations of clinician experiences in using newly introduced tools or design studies that evaluated experiences with tools while they were under development. These studies are valuable but can provide only limited insights into the impact of the long-term use of tools on experiences, job satisfaction, and workflows. This suggests that 1 factor that may be relevant in driving the small size of the research literature on this topic is poor alignment with research agendas and funding priorities.

Among the studies that were included in the review, we also observed that although the moderators that might positively or negatively affect the use of digital tools and their outcomes were commonly reported, the experiences of HCPs, such as their thoughts, emotions, and feelings, were less frequently discussed in the literature. However, these direct experiences are likely to have a significant impact on the well-being of clinicians, the care they can provide patients, and the overall functioning of the health care system. This suggests that research specifically targeting the direct lived experiences of clinicians using digital tools in hospital settings would benefit from an explicit emphasis on individual thoughts and emotions as an important driver for HCPs to use digital tools.

Digital tools may enforce or be the front end for administrative tasks, taking time away from the work that clinicians want to do. Administrative tasks are typically seen as less meaningful work, and finding meaning in one’s work serves to offset stress and reduce burnout [78].

Another significant aspect is workflows with interruptions and higher cognitive burden, which contribute to lower clinician satisfaction and higher emotional exhaustion. This is evident in previous studies that reported that the introduction of EHRs resulted in numerous additional and often unnecessary interruptions caused by excessive and often irrelevant or poorly timed alerts and inbox notifications that disrupt the workflows and interactions with patients [79,80]. Such interruptions have been identified as a major issue contributing to alert fatigue and are likely to be associated with burnout [81,82]. Furthermore, previous studies have highlighted information overload as a serious problem associated with the use of EHR that also contributes to this problem [83,84]. The findings suggest that a digital tool should strike a balance between reducing workload and promoting critical thinking among HCPs when dealing with provided information.

The usability and interoperability problems with the EHR, combined with the demands of documentation and reporting requirements, create an administrative and clerical burden for clinicians that allows less time for patient care or nonwork-related activities. This is exemplified in an observational study of 57 physicians in 4 specialties, where physicians dedicated 49.2% of their office day to EHR and desk work and 37% during examination room visits, nearly double the amount of time spent doing direct patient care tasks. In addition, physicians reported spending 1 to 2 hours of after-hours work, primarily focused on EHR tasks [85,86].

This also affects nurses and nursing leaders, who are often frustrated with the current EHR system, as its design fails to support their workflows and presents significant usability issues. This not only impacts nurses themselves but also has negative repercussions on patients and health care organizations [87]. Another study indicated that nurses spend up to half of their time in front of a computer documenting patient information [88].

The digitalization of clinical work not only allows for the capturing of documentation in digital systems but also enables the possibility or expectation of doing so remotely and from home. In this sense, digitalization in hospital settings mirrors a wider transformation of the workplace that is ongoing and has been accelerated by the recent pandemic. Our findings suggest that clinicians report some positive outcomes from the use of digital tools, including improved quality of patient care, enhanced workflow efficiency, and better information availability. In contrast, negative outcomes such as increased workload, heightened patient safety risks, outdated or missing information, and disruptions in workflow were also identified as still relevant, even with modern clinical information systems. The positive and negative outcomes were often perceived in pairs, such as increased patient safety versus increased patient safety risks, better information availability versus missing or outdated information, increased workflow efficiency versus complications, and workflow interruptions.

The findings of our review suggest that the use of digital tools by clinicians can be influenced by various moderators. These moderators can positively enhance the use of digital tools. For instance, adequate training may equip clinicians with the essential skills and confidence to effectively use digital tools, along with seamless workflow integration, a user-friendly interface design, and favorable organizational structures. This ensures minimal disruption and efficient use and makes it easier for clinicians to navigate the digital tools. Conversely, certain moderators can have negative effects on the use of digital tools, such as unfavorable organizational structures, leading to a lack of support and motivation; inadequate training, which may lead to frustration, errors, or misuse of the tool; and insufficient interface design and customization, which may lead to struggles while navigating the interface or finding the desired information need. As with outcomes, positive and negative moderators are frequently reported as opposing pairs, as is the case with sufficient training positively impacting tool use and lack of training hindering tool use, similar to favorable and unfavorable organizational structures.

### Limitations

This review encompasses a diverse range of studies in hospital settings, and the underlying theoretical framework highlights the complexity of the interconnection between positive and negative experiences, moderators, and outcomes.

This review has several limitations. Although every effort was made to be comprehensive in the search for relevant literature, it is possible that the inclusion and exclusion criteria may have biased the results. The review focused solely on physicians and nurses working in a hospital setting, either secondary, tertiary, or quaternary care, and not in primary care. In addition, we did not include studies that were focusing on pilot, implementation, or validation studies. As we were primarily interested in the experience of HCPs using digital tools, we also did not focus on studies that evaluated the improvement of quality of care as a primary study outcome. As a result, some papers exploring the relevant experiences of general practitioners and in other study contexts were excluded. We also excluded studies that involved populations of students who had not yet started their professional careers.

Although our search was conducted using global research repositories, the focus on English language publications may have biased the results; indeed, a majority of the included studies were conducted in English-speaking countries.

Furthermore, owing to the timing of our systematic review, experiences of clinicians using large language models such as ChatGPT have not yet been reported in the literature we reviewed. However, this is likely to be an increasingly important topic for future research.

### Implications for Future Research

This review indicates a need for future studies to focus more on the direct lived experiences of HCPs including thought processes, feelings, and emotions, as this has not been widely reported in previous studies. Moreover, there is a need to explore the experiences of HCPs in other regions of the world where digital transformation, drivers, constraints, workflows, and organizational cultures may differ markedly from those reflected in the predominant body of the existing literature. For example, a notable research gap exists in various regions, including South America; significant parts of Africa, Southeast Asia, and the Pacific; as well as in specific countries within Middle and Eastern Europe (Figure 2). Only limited attention has been directed toward exploring this topic in these regions.

### Conclusions

This literature review surveyed the recent experiences of clinicians using digital tools in a hospital setting. This paper presents information about the experiences as well as moderators that can promote or hinder the use, and outcomes of digital tools in hospitals and identifies opportunities for further research. We proposed a theoretical framework to explain the complex interplay between the use of digital tools, experience, moderators, and outcomes. The framework emphasized the need to consider the individual experiences of users, which can be influenced by either the outcome of using digital tools or by the use of the tool itself. In addition, our review also revealed that tool-specific factors, such as the design and goals of the tool, as well as the professional role and responsibilities can impact the user experiences. The review findings highlight the influence of adequate training for clinicians using digital tools and emphasize the need for favorable organizational structures to positively influence use.

## Appendix

Multimedia Appendix 1 PRISMA (Preferred Reporting Items for Systematic Reviews and Meta-Analyses) checklist.

Multimedia Appendix 2 Search strategy.

Multimedia Appendix 3 Inclusion and exclusion criteria.

Multimedia Appendix 4 Data extraction and quality assessment template.

Multimedia Appendix 5 Identified framework annotations within and across publications, digital tools, and study populations.

## Abbreviations

AI: artificial intelligence
CDS: clinical decision support
EHR: electronic health record
HCP: health care professional
PRISMA: Preferred Reporting Items for Systematic Reviews and Meta-Analyses
