# Supplementary material for: Experience of Health Care Professionals Using Digital Tools in the Hospital: Qualitative Systematic Review
Source: JMIR Hum Factors. 2023 Oct 17;10:e50357. doi: 10.2196/50357 (PMC10618886; doi:10.2196/50357)
Supplement: Multimedia Appendix 2 [file humanfactors_v10i1e50357_app2.docx]

**Multimedia Appendix 2: Search strategy and information sources**

The overall search strategy is based on keywords related to “digital”, “health care professionals”, “experience” and “qualitative”.

**Table S1.** Search strategies.

| **Database** | **Search string** |
| --- | --- |
|  |  |
| PubMed | (“digital tool*” OR “digital application” OR “digital device*” OR “digital health technolog*” OR “technologic tool*” OR “technologic application*” OR “electronic knowledge” OR “electronic patient record*” OR “electronic medical record*” OR “AI tool*” OR “decision support system*” OR “decision-support system*” OR “decision support tool*” OR “decision-support tool*”) AND (“health care professional*” OR “healthcare professional*” OR HCP* OR clinician* OR physician* OR doctor* OR practitioner* OR specialist* OR nurse* OR “medical staff” OR “clinical staff”) AND (experience* OR expectation* OR implement* OR perception* OR adoption OR acceptance OR usability OR preference*) AND qualitative* |
| Scopus | TITLE-ABS-KEY ( ( "digital tool*" OR "digital application" OR "digital device*" OR "digital health technolog*" OR "technologic tool*" OR "technologic application*" OR "electronic knowledge" OR "electronic patient record*" OR "electronic medical record*" OR "AI tool*" OR "decision support system*" OR "decision-support system*" OR "decision support tool*" OR "decision-support tool*" ) AND ( "health care professional*" OR "healthcare professional*" OR hcp* OR clinician* OR physician* OR doctor* OR practitioner* OR specialist* OR nurse* OR "medical staff" OR "clinical staff" ) AND ( experience* OR expectation* OR implement* OR perception* OR adoption OR acceptance OR usability OR preference* ) AND qualitative* ) |
| Web of Science | (TS=((“digital tool*” OR “digital application” OR “digital device*” OR “digital health technolog*” OR “technologic tool*” OR “technologic application*” OR “electronic knowledge” OR “electronic patient record*” OR “electronic medical record*” OR “AI tool*” OR “decision support system*” OR “decision-support system*” OR “decision support tool*” OR “decision-support tool*”) AND (“health care professional*” OR “healthcare professional*” OR HCP* OR clinician* OR physician* OR doctor* OR practitioner* OR specialist* OR nurse* OR “medical staff” OR “clinical staff”) AND (experience* OR expectation* OR implement* OR perception* OR adoption OR acceptance OR usability OR preference*) AND qualitative*) |
