# Supplementary material for: Experience of Health Care Professionals Using Digital Tools in the Hospital: Qualitative Systematic Review
Source: JMIR Hum Factors. 2023 Oct 17;10:e50357. doi: 10.2196/50357 (PMC10618886; doi:10.2196/50357)
Supplement: Multimedia Appendix 3 [file humanfactors_v10i1e50357_app3.docx]

**Multimedia Appendix 3: Eligibility criteria**

**Table S1.** Inclusion and exclusion criteria for literature.

| **Inclusion criteria** | **Exclusion criteria** |
| --- | --- |
|  |  |
| Qualitative, descriptive interview studies using a defined qualitative approach and semi-structured interviews presenting distinct qualitative data and results in order to capture firsthand experiences reported in healthcare professional’s own words | Quantitative studies and analyses |
| Studies in English language | Studies not in English language |
| Studies published from 01.01.2018 - 01.01.2023 | Studies outside the time frame |
| Studies that describe the experiences of HCPs using digital tools in a hospital setting with at least 6 months of experience | Studies focusing on a different population, e.g., students or health care professionals in primary care, e.g., general practitioners |
| Qualitative components of mixed methods studies | Hypothetical studies  Survey studies  (Systematic) reviews |
| Peer-reviewed studies | Conference abstracts  Dissertations and theses |
|  |  |
