# Supplementary material for: Experience of Health Care Professionals Using Digital Tools in the Hospital: Qualitative Systematic Review
Source: JMIR Hum Factors. 2023 Oct 17;10:e50357. doi: 10.2196/50357 (PMC10618886; doi:10.2196/50357)
Supplement: Multimedia Appendix 4 [file humanfactors_v10i1e50357_app4.docx]

**Multimedia Appendix 4: Data extraction and quality assessment template**

**Table S1.** Data extraction template.

| **Section** | **Characteristics** |
| --- | --- |
|  |  |
| General publication information | - Publication title - Authors - Study ID (DOI) - Publication year - Publication origin (based on corresponding author) |
| Key study and method characteristics | - Aim of study - Study location/s (country(s) in which the study was conducted) - Study methodology/design (e.g., interview study, multi-method study) - Study recruitment - Sampling strategy - Theory/Framework used - Study settings - Population - Number of participants (number of interview participants, either physicians or nurses) |
| Study population and background characteristics | - Location(s) of employment - Medical background / Main area(s) of expertise - Gender - Age - Total work experience - Experience in working with digital tools - Type of digital tool/s used/assessed |
| Key findings | - Primary outcomes: identified themes based on healthcare professional’s experience using digital tools - Secondary outcomes, if applicable |

**Table S2.** Quality assessment template.

| **Section** | **Questions** |
| --- | --- |
|  |  |
| Result validity | - Was there a clear statement of the aims of the research? - Is a qualitative methodology appropriate? |
| Research design and methods | - Was the research design appropriate to address the aims of the research? - Was the recruitment strategy appropriate to the aims of the research? - Was the data collected in a way that addressed the research issue? - Has the relationship between the researcher and participants been adequately considered? |
| Sufficiency of results | - Have ethical issues been taken into consideration? - Was the data analysis sufficiently rigorous? - Is there a clear statement of findings? |
| Added value | - How valuable is the research? - Will the results help locally? |
