# Supplementary figures and images for: Experience of Health Care Professionals Using Digital Tools in the Hospital: Qualitative Systematic Review
Source: JMIR Hum Factors. 2023 Oct 17;10:e50357. doi: 10.2196/50357 (PMC10618886; doi:10.2196/50357)

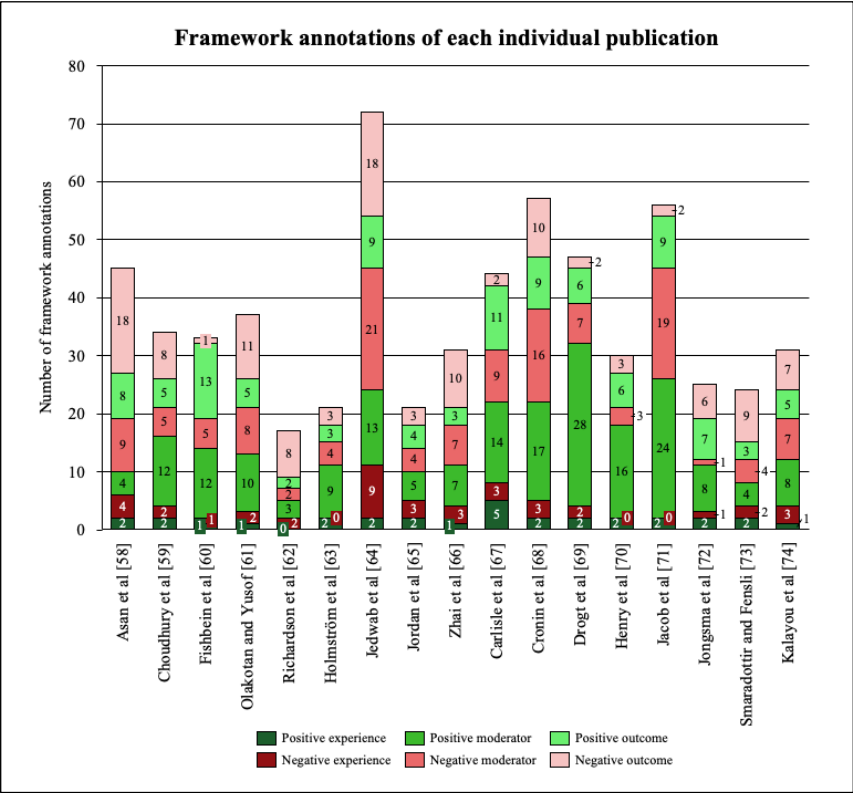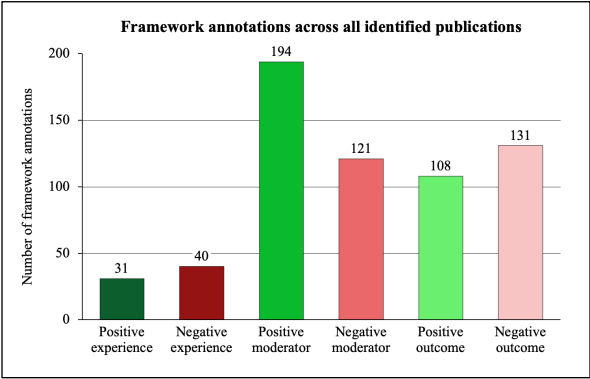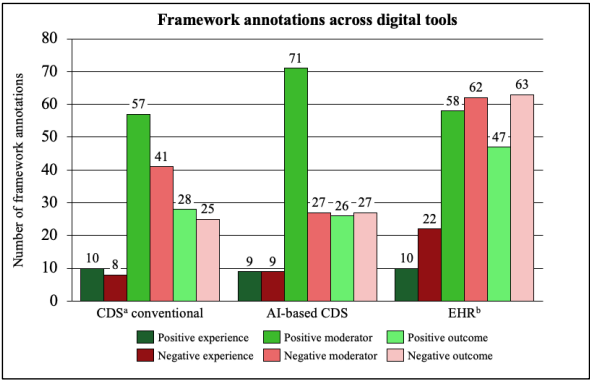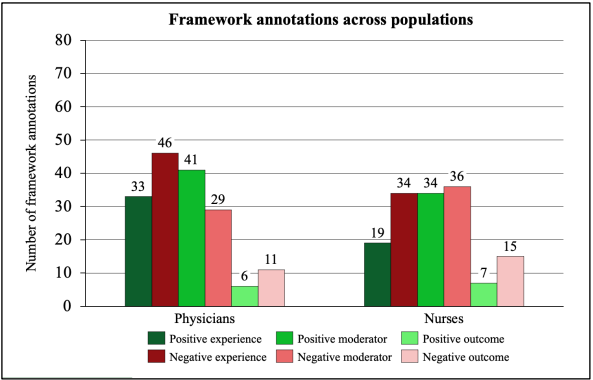

<sup>a</sup>CDS: clinical decision support.

<sup>b</sup>EHR: electronic health record.

Supplement: Multimedia Appendix 5 [file humanfactors_v10i1e50357_app5.pdf]
